# Toward Personalized Salbutamol Therapy: Validating Virtual Patient-Derived Population Pharmacokinetic Model with Real-World Data

**DOI:** 10.3390/pharmaceutics16070881

**Published:** 2024-06-30

**Authors:** Lara Marques, Nuno Vale

**Affiliations:** 1 PerMed Research Group, Center for Health Technology and Services Research (CINTESIS), Rua Doutor Plácido da Costa, 4200-450 Porto, Portugal; lara.marques2010@hotmail.com; 2 CINTESIS@RISE, Faculty of Medicine, University of Porto, Al. Prof. Hernâni Monteiro, 4200-319 Porto, Portugal; 3 Department of Community Medicine, Health Information and Decision (MEDCIDS), Faculty of Medicine, University of Porto, Rua Doutor Plácido da Costa, 4200-450 Porto, Portugal

**Keywords:** interindividual variability, popPK modeling, PBPK modeling, salbutamol, pharmacokinetics, virtual data patients

## Abstract

Interindividual variability, influenced by patient-specific factors including age, weight, gender, race, and genetics, among others, contributes to variations in therapeutic response. Population pharmacokinetic (popPK) modeling is an essential tool for pinpointing measurable factors affecting dose-concentration relationships and tailoring dosage regimens to individual patients. Herein, we developed a popPK model for salbutamol, a short-acting β_2_-agonist (SABA) used in asthma treatment, to identify key patient characteristics that influence treatment response. To do so, synthetic data from physiologically-based pharmacokinetic (PBPK) models was employed, followed by an external validation using real patient data derived from an equivalent study. Thirty-two virtual patients were included in this study. A two-compartment model, with first-order absorption (no delay), and linear elimination best fitted our data, according to diagnostic plots and selection criteria. External validation demonstrated a strong agreement between individual predicted and observed values. The incorporation of covariates into the basic structural model identified a significant impact of age on clearance (Cl) and intercompartmental clearance (Q); gender on Cl and the constant rate of absorption (ka); race on Cl; and weight on Cl in the volume of distribution of the peripheral compartment (V2). This study addresses critical challenges in popPK modeling, particularly data scarcity, incompleteness, and homogeneity, in traditional clinical trials, by leveraging synthetic data from PBPK modeling. Significant associations between individual characteristics and salbutamol’s PK parameters, here uncovered, highlight the importance of personalized therapeutic regimens for optimal treatment outcomes.

## 1. Introduction

Therapeutic response varies considerably among patients, with drugs being pharmacologically effective in some while ineffective or even toxic in others [1,2]. The precise determination of a safe and effective drug dose is directly dependent on understanding the PK and PD properties of that particular drug [3]. Indeed, PK variability plays a crucial role in the success of drug treatments. Patient-specific factors such as age, weight, body mass index (BMI), gender, hormonal status, race, ethnicity, renal and hepatic function, genetic polymorphisms, disease status, concomitant therapies, smoking, and dietary habits can all contribute to variation in drug disposition [1,4].

As pharmacometrics advances as a cornerstone of precision medicine, there is a growing interest in understanding the impact of these factors on the PK profile of drugs. Thus, population pharmacokinetic (popPK) modeling has been recognized as an essential tool for accurately identifying measurable pathophysiological factors that influence the dose–concentration relationship, allowing for the optimization of dosage regimens tailored to individual patients, thereby achieving improved therapeutic outcomes [5]. It is evident that the careful and accurate collection of data during drug development and subsequent analyses is extremely useful for modeling PK characteristics [6,7].

Indeed, PK variability is present in all drugs. Within the realm of treating prevalent chronic diseases such as asthma, it becomes crucial to understand the impact of individual patient characteristics on these drugs [8]. In particular, salbutamol, a short-acting β_2_-agonist (SABA), has been used as an alternative symptomatic reliever for bronchoconstriction events [9,10,11]. This phenomenon is effectively mitigated by targeting β_2_-adrenergic receptors in the smooth muscle of the airways, leading to bronchodilation. As a result, the airways widen, allowing for improved airflow in and out of the lungs. When salbutamol is inhaled, immediate relief is experienced due to its rapid onset of action [12,13,14,15]. For this reason, the use of SABAs on an “as-needed” basis has moved towards regular use, regardless of prevailing symptoms, resulting in their overuse. However, evidence linking regular SABA use with an increased risk of exacerbations due to β_2_-receptor downregulation, loss of bronchodilator response, and increased airway inflammation has prompted a significant shift in asthma management [16,17,18,19,20]. Since 2019, the Global Initiative for Asthma (GINA) no longer recommends monotherapy with SABA [21]. Currently, salbutamol is administered either in combination with inhaled corticosteroids (ICSs) or as an alternative reliever. However, despite being on a smaller scale, SABA alone is still prescribed, particularly salbutamol [22,23]. In this context, studying variations in salbutamol PK becomes essential to clarify the SABA therapeutic landscape beyond the molecular level.

Progress in computational science has positioned in silico studies as an important and well-recognized methodology in drug discovery, development processes, and optimization of therapeutic regimens post-market integration. Understanding the influence of different patient characteristics is important for individualizing and personalizing treatment, allowing for maximum efficacy while reducing potential adverse effects. Coupled with this, in silico studies offer numerous advantages in terms of cost and sustainable research, as they can reduce the use of animal models and allow for more efficient clinical trial design [24]. Human trials are already being partly replaced by in silico trials [25,26]. The European Medicines Agency (EMA) and the Food and Drug Administration (FDA) are both endorsing these studies and have been directing efforts towards the development of computational simulations [27,28,29]. Concerning the optimization of therapeutic regimens considering interindividual variability, popPK models are the easier and less expensive way to achieve this [30]. 

The delivery of drugs via inhalation is affected by a wide range of factors, in particular physiological and pathological variables [31]. To our knowledge, there are no studies of this nature on salbutamol. Thus, developing a popPK study to explore the impact of patient variability leads to a comprehensive understanding of how salbutamol is absorbed, distributed, metabolized, and excreted within a diverse population.

Our study aimed to develop a popPK model of salbutamol administered via dry-powder inhaler (DPI, one of the most common formulations available) and to identify which individual characteristics benefit from the current therapeutic regimen. Additionally, we aimed to demonstrate the feasibility of developing a popPK model using a set of synthetic data generated from physiologically-based pharmacokinetic (PBPK) models. To achieve this, the model derived from virtual patients will be externally validated using clinical data from an equivalent study conducted in real patients. We hypothesize that the popPK model developed with virtual patients will exhibit a predictive performance comparable to models derived from traditional clinical trials, highlighting the potential of virtual patient modeling in advancing this field.

## 2. Materials and Methods

### 2.1. Virtual Patient Data Collection

The physicochemical and PK properties of salbutamol were estimated using ADMET Predictor^®^ (Version 10.4; Simulation Plus Inc., Lancaster, CA, USA). Its chemical structure was drawn in MedChem Designer (Version 5.5; Simulation Plus Inc., Lancaster, CA, USA), according to its SMILES, and then imported into ADMET Predictor^®^. 

PBPK models developed in GastroPlus software (Version 9.8.3; Simulation Plus Inc., Lancaster, CA, USA) were used to generate virtual patient PK data. These models were built using the values predicted by ADMET Predictor^®^. All patients were virtually treated with 600 μg of salbutamol DPI (3 successive inhalations of 200 μg). Thus, the dosage form selected in the software was PL:IT powder, which represents the delivery of inhaled drug in solid phase, as a single dose. The PK profile of salbutamol was modeled in different PBPK models with specific individual characteristics, namely, age, weight, race, and gender. In particular, subjects aged 5, 10, 20, 30, and 65 years were included. The body mass index (BMI) scale (BMI of 18.5–24.9 is normal, BMI of 25–29.9 is overweight, and BMI ≥ 30 is obese) was employed to determine weight. American, Japanese, and Chinese race groups were examined in this study. All patients were healthy, meaning that they had no adjacent pathological conditions. Detailed characteristics of these individuals are summarized in Supplemental Table S1.

Parameters such as bioavailability (Fa, fraction absorbed; FDp, fraction of the drug concentration in the portal vein; and F, fraction of the drug concentration in blood), maximum plasma concentration (*C_max_*), time required to maximum plasma concentration (*T_max_*), area under the curve (AUC), and maximum concentration in the liver (*C_max Liver_*), were derived from ADMET Predictor^®^. The drug disposition-based parameters were simulated in the virtual patients during 12 h. Quantitative and visual (plots) outputs of the drug concentration profile were extracted to establish a dataset for the subsequent phase of the study. 

All PK parameter values were compared with data from the literature, and the plasma concentration profile plots were visually inspected to evaluate and validate all PBPK models.

### 2.2. Noncompartmental Analysis of PK Data

To establish initial PK metrics, a noncompartmental analysis (NCA) of the virtual dataset was performed in PKAnalix 2023R1 (Lixoft, Antony, France). The integral method employed was linear trapezoidal linear, with equal weighting assigned to each data point. As an acceptance criterion, we utilized adjusted R^2^ to select the number of points for the terminal phase, enabling the determination of the slope of the linear regression for calculating λ_z_ (elimination rate constant). Subsequently, all PK parameters were determined.

### 2.3. Population Pharmacokinetic Modeling

The virtual salbutamol data were inputted to build a nonlinear mixed-effects model (NLME) through estimation by maximum likelihood using the stochastic approximation expectation-maximization (SAEM) algorithm in Monolix Suite 2023R1 (Lixoft, Antony, France). The determination of conditional means and standard deviations for individual population PK parameters involved the utilization of Markov chain Monte Carlo (MCMC) convergence assessment. The objective function value (OFV), expressed as −2 × log likelihood (−2LL), and the Akaike information criterion (AIC) and Bayesian information criterion (BIC), were determined to select the best popPK model. Significant reductions in these parameters indicated better model fitting.

#### Structural and Statistical Models

Several PK models for extravascular administered salbutamol were investigated (one-, two-, or three-compartment models with first- or zero-order absorption with/without lag time or transit and with linear or Michaelis–Menten elimination). PK models correspond to a system of ordinary differential equations (ODEs) which describes transfers between compartments and elimination from the central compartment. For the observation model, the default combined 1 error model (additive and proportional term) were initially applied. For the individual model, we also keep the default: log-normal distributions for PK parameters. In a preliminary analysis, we ignored the inclusion of covariates in the popPK models. The optimal combination of structural and statistical models was systematically selected by evaluating the OFV, model diagnostics, and ensuring biologically plausible and accurately population mean estimates (as measured by relative standard error (RSE)).

### 2.4. Model Evaluation and Covariate Selection

After running several popPK models, the assessment of the model fitting was based on various evaluative metrics. BIC, the precision of estimates (RSE), and the goodness-of-fit (GOF) allowed the final decision for selecting the best popPK model. The model with the lowest corrected BIC (BICc) value was selected. In addition, complementary criteria including AIC, to estimate the model quality, and OFV, for identifying smaller values indicating superior fit, were also employed. The estimated population parameters’ standard errors and the random effects error models’ standard errors were also computed. Further, several diagnostic plots were used to visually test the model’s fit, including observation vs. individual predictions (IPREDs), population-weighted residuals (PWRESs) vs. time/predictions, individual weighted residuals (IWRESs) vs. time/predictions, and the distribution of the empirical and theoretical standard Gaussian probability density functions (PDFs) against IWRESs and normalized prediction distribution errors (NPDEs). A visual predictive check (VPC) using a 90% prediction interval was performed to graphically assess misspecifications in structural, variability, and covariate models. 

Age, height, and weight were the continuous independent covariates tested to be included in the popPK model. Gender and race were designated as categorical covariates. The selection of covariates was based on the model proposed by the software program. Using the ANOVA statistical test for the categorical covariate and the Pearson’s correlation test for the continuous covariate, a *p*-value can be calculated. Regardless of their incorporation into the model, the random effect–covariate associations are sorted using the *p*-values. The forward and backward method was employed to select covariates. Until there are no correlation *p*-values over a threshold, the covariate with the smallest correlation *p*-value is included in the model, or the next smallest if the smallest has already been attempted. Until there are no correlation *p*-values below a threshold, the covariate with the highest correlation *p*-value is disregarded, or the next highest if the highest value has already been attempted. Therefore, covariates with a *p*-value less than 0.05 that improved the fit while lowering BIC remained in the model.

For the evaluation of the proposed final model, we employed statistical tests using individual parameters drawn from the conditional distribution. Correlation and Wald test were used to assess whether covariates should be removed from the model. The normal distribution of random effects and the symmetry around 0 of residuals’ distribution were examined through Shapiro–Wilk test and symmetry test, respectively.

### 2.5. External Validation

External validation was conducted using data from an open-label, randomized, crossover, two-cohort, single-dose study in healthy volunteers to evaluate the unit dose dry powder inhaler (UD-DPI) for the delivery of salbutamol and to compare the PK profile with the MDI and Diskus presentations (NCT01984086) [27], with access granted by GlaxoSmithKline (GSK). The focus of data extraction was on Part A of the study, encompassing 30 individuals subjected to Treatment A. This treatment involved a single administration of salbutamol (as sulfate) (200 μg per blister of 1.6% blend), delivered via UD-DPI through the inhalation of 3 blisters, resulting in a cumulative dose of 600 μg. The selection of this treatment aimed to align with the dose administered in the development of the popPK model using virtual patients. 

The study cohort comprised healthy nonsmoking male/female individuals, aged 18 to 65 years, with a body weight ≥ 50 kg, and a body mass index (BMI) within the range of 19.0 to 34.0 kg/m^2^. Participants had to provide written informed consent. Main exclusion criteria included current or history of chronic liver disease, a history of salbutamol sensitivity, positive tests for Hepatitis B, Hepatitis C, and HIV, pregnancy, breastfeeding, or active attempts to conceive. Further details can be found in the study protocol. 

Collected data included demographic information (age, race, gender, weight, and height) and details about plasma concentrations. Salbutamol PK analysis involved blood samples collected at the following time points: 0, 0.08, 0.17, 0.33, 0.50, 0.75, 1, 1.5, 2, 4, 6, 8, 10, and 12 h. Plasma concentrations of salbutamol were quantified using an approved bioanalytical method.

**Figure 1:**
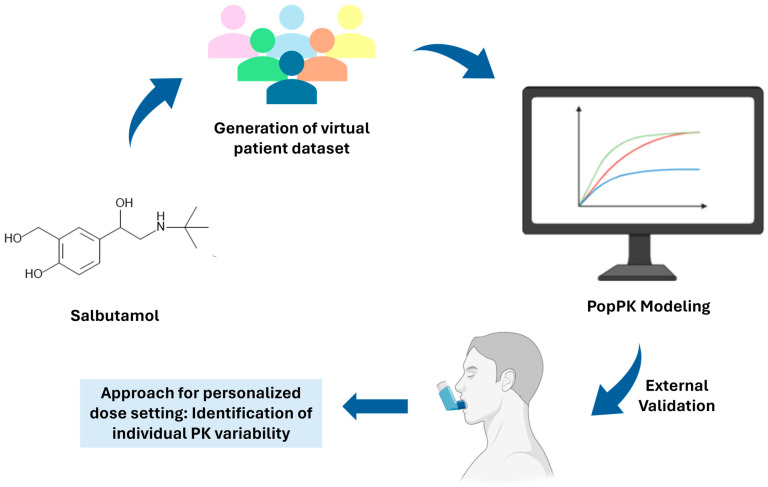
Overview of the workflow popPK modeling.

Upon acquisition of the dataset for input into Monolix software, the popPK model derived from virtual data was applied, following the previously outlined steps. The validation of the virtual data-based model was performed through visual inspection of outcomes after applying the same popPK model to the clinical dataset. A general scheme of the workflow is shown in Figure 1.

## 3. Results and Discussion

During the process of developing a precise popPK model, pharmacometricians encounter significant challenges, particularly regarding the substantial quantity and access to PK data and personal information required, due to several limitations imposed by company policies and ethical considerations. In addition, data derived from clinical trials are often incomplete, especially datasets collected in large, late-phase trials or during routine healthcare or follow-up visits. Most of these data may be missing or inaccurate due to a variety of factors such as study site noncompliance, patient noncompliance, inappropriate sample handling, data entry errors, and analytical issues. Consequently, how these missing or erroneous data are handled can impact their interpretation. Although popPK modeling requires a smaller volume of data points, that is, it is possible to develop an accurate model with sparse data (few observations per subject) [30], sometimes even this is difficult to obtain in clinical studies (especially in Phase I trials), because volunteers have to undergo a number of blood draws, raising serious ethical questions, especially in special populations such as children. Indeed, there are some guidelines that help pharmacometricians in dealing with these challenging data, but the truth is that this will always have a significant impact on the final model and outcomes [32,33,34]. 

In this context, PK data for salbutamol are scarce, as we previously stated [35], and to circumvent all the aforementioned issues, our study proposes a new way to study therapeutic regimen optimization through the generation of a synthetic dataset, which encompasses a diverse set of virtual patients. This approach has already been employed in recent studies and involves the artificial generation of data that mimics real-world data [36,37,38,39]. For example, artificial intelligence (AI) algorithms have been proposed in this scope. In the context of our study, a virtual dataset was generated using PBPK models to simulate salbutamol PK profiles across various demographic characteristics.

### 3.1. Demographic Characteristics

Thirty-two virtual subjects were included in this study. Among these subjects, there are 18 males and 14 females, with a median age of 20.0 years and a BMI median of 21.3 kg/m^2^. Furthermore, the virtual population is composed of two groups, according to race: American Indian or Alaskan Native represent 38% of total patients, with East Asian (Japanese and Chinese) patients representing 62% of the sample. These demographic characteristics are summarized in Table 1.

Notable differences across various parameters are observed between virtual patients and patients enrolled in the clinical study. The mean age is 26.8 years. Gender distribution varies, with virtual patients having a more balanced ratio, while clinical study patients skew towards a higher percentage of males (73%). In terms of body composition, virtual patients, on average, have a lower BMI, height, and weight. This is attributed to the inclusion of Japanese and Chinese individuals in the virtual dataset, who inherently present lower values. Consequently, racial composition significantly differs between the two datasets. Virtual patients are predominantly East Asian or American, whereas patients included in the clinical study are mostly Caucasian. In fact, studies of this nature do not accurately represent the “real-world” population [40]. Clinical trials often impose stringent criteria to ensure sample homogeneity and minimize confounding variables, employing limited analyses tailored to address specific research questions. Further, voluntary participation introduces a potential bias, as participants are self-selected and willing to engage. Additionally, due to resource constraints, many studies exhibit limited representation of racial and ethnic groups, resulting in underrepresentation or inadequate portrayal of population diversity, creating a gap between the trial world and the real world [41,42,43,44].

Efforts within the scientific community have been undertaken to address this gap by enhancing diversity in clinical trial recruitment [40]. Therefore, this virtual dataset aims to present a more diverse ethnic group. Ideally, it would include Caucasians as well; however, the PBPK modeling software mimicking the patient cohort has limitations concerning ethnic groups. 

Subsequently, an NCA of both datasets was conducted, allowing the collection of PK parameters to compare their distribution between virtual and real populations. Table 2 displays the *C_max_*, AUC, *T_max_*, and the volume of plasma from which drug is removed from the human body (C*l*). The observed differences in values may be attributable to variations in the characteristics of the datasets.

**Figure 2:**
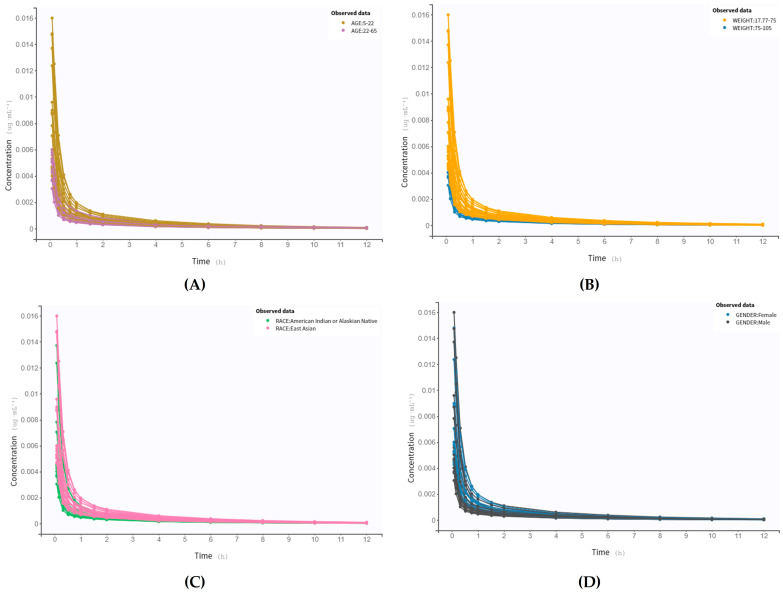
Observed data of plasma salbutamol concentration (μg/mL) through time (h), stratified by (**A**) age, (**B**) weight, (**C**) race, and (**D**) gender. The absorption phase cannot be visualized.

The exploration of virtual patient data is depicted in Figure 2. The data were stratified based on some crucial individual characteristics: age, weight, race, and gender. A noticeable difference is observed between the younger and the adult and elderly populations—younger subjects exhibit higher concentrations of salbutamol over time. The pediatric population is recognized as a special group for drug therapy, as many physiological changes occurring at these ages have a significant impact on the PK of several compounds. Factors such as pH, differences in bile luminal concentrations, intestinal permeability, and variations in enzymatic activity may explain higher plasma concentrations of salbutamol in younger individuals [45]. Consequently, toxicity may occur in this age range, highlighting the importance of dose adjustment in children. In the context of this study, the dose administered to virtual patients under 18 years is equivalent to the dose for children (up to 100–200 μg as a single dose on demand) [46].

Weight also influences the PK profile of this drug, with individuals with more than 75 kg (overweight and obese) displaying reduced values. Regarding race and gender, with this preliminary analysis, it is not visually discernible to determine the impact that different groups may have on the kinetics of this β-agonist.

Data exploration also uncovered several correlations between PK parameters and covariates. There is a negative correlation between the AUC and both height and weight, implying that individuals with greater height or weight tend to have lower overall exposure to the drug over time. Similarly, a negative correlation was observed between C*_max_* and height/weight. On the other hand, there is a positive correlation between drug clearance and height or weight. Individuals with higher height or weight tend to eliminate the drug from their bodies at a faster rate.

An interesting observation is that Asians exhibit higher AUC and C*_max_* values and lower clearance values compared to the American population, suggesting that, on average, Asians may experience higher overall drug exposure and slower elimination of the drug from their bodies. 

### 3.2. Final popPK Model

The plasma concentration–time profiles of inhaled salbutamol in DPI form were best described by a two-compartment model, with first-order absorption (no lag time), and linear elimination. During the development of the structural model, several models were created and evaluated based on criteria detailed in Section 2.3. The model with the lowest BICc value, following visual inspection of the VPC, was selected. BICc values for each tested model are provided in the Supplementary Material (Table S2). In particular, for this model, a −2LL of −6480.18 and a BICc of −6420.66 were obtained.

Therefore, the most suitable model for this dataset included first-order absorption, as salbutamol is absorbed through passive diffusion [47], a common mechanism in drugs following first-order kinetics, without lag time, considering salbutamol is inhaled and has an immediate onset of action at its target. Two compartments were also incorporated, a central compartment and a peripheral compartment, although the literature reports a one-compartment model to describe salbutamol PK. In fact, after inhalation of salbutamol, a significant portion of the dose is swallowed, and only a small fraction (around 20%) reaches the specific target (lungs) [48]. Thus, the inclusion of two compartments in the PK model may be logical, with the central compartment being the lungs, where drug distribution occurs, binding to the β_2_-adrenergic receptors present in lung tissue, and the peripheral compartment being the gut, where the drug is delivered due to swallowing that occurs in inhaled drugs. Additionally, the most appropriate elimination type is linear elimination, which means that the elimination rate is directly proportional to the drug concentration. 

Covariate analysis, employing the model proposed by Monolix^®^, encompassed gender, age, height, weight, and racial population. The incorporation of all covariates significantly improved the base structural model. Therefore, we highlighted the influence of individual patient characteristics on salbutamol’s kinetics: age exhibited a significant impact on C*l* and intercompartmental clearance (Q); gender influences absorption constant rate (k_a_) and C*l*; race has an impact on C*l*, and weight showed a considerable influence on C*l*, Q, and on the volume of distribution of peripheral compartment (V2). The interindividual variability (IIV) in these PK parameters was considered by applying a normal distribution and a combined residual model, consisting of proportional and additive terms. Parameter estimates for the final structural model are shown in Table 3. All structure parameters were estimated with good precision, with relative standard error (RSE) < 30% for fixed effects, except for the volume of distribution of the central compartment (V1), which exhibited an infinitely large standard error. Regarding the RSE of random effects, there is insufficient accuracy in some estimates. A correlation between k_a_ and Q was identified from scatter plots for each pair of random effects, along with the Pearson correlation coefficient. This correlation was introduced into the model to achieve a more accurate estimation of these parameters.

#### Model Assessment Using the Diagnostic Plots

The low *p*-values (*p* < 0.05) in Pearson’s correlation test confirmed that the correlation between parameters and covariates was significant. These tests were in agreement with Wald test results, where low *p*-values (*p* < 0.05) indicated the relevance of covariate effects.

**Figure 3:**
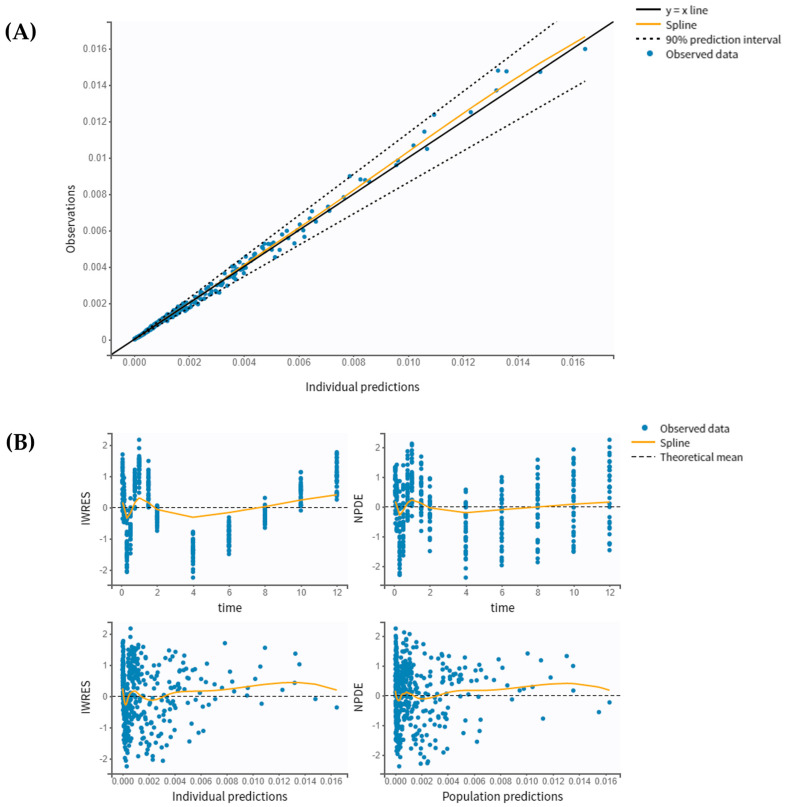
Goodness-of-fit plots of the final popPK model: (**A**) Observed salbutamol concentration vs. individual predictions using conditional mode (EBEs); and (**B**) IWRES vs. time and individual predicted, and NPDE vs. time and population predicted.

**Figure 4:**
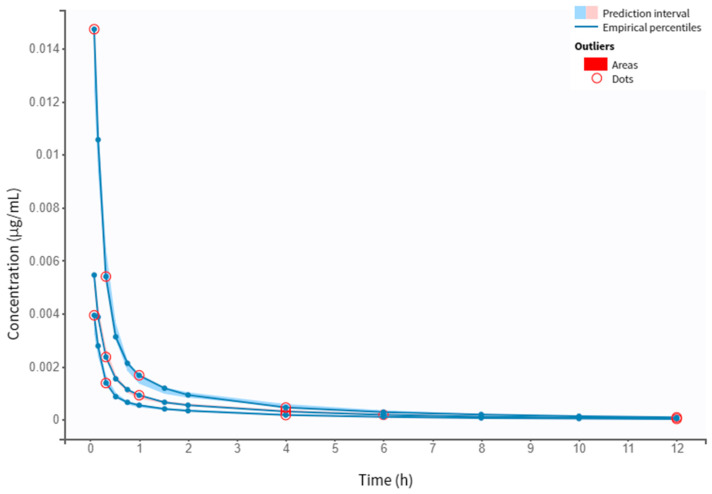
VPC for the two-compartment model with first-order absorption, no delay, and linear elimination. The VPC of the final covariate PK model displays the prediction intervals for the 10th, 50th, and 90th percentiles (shaded areas, from bottom to top, respectively). Outliers are visualized as red dots and areas.

The goodness-of-fit plots of the final PK model with covariates are shown in Figure 3. The individual predicted values, obtained through the conditional model (empirical Bayes estimates, EBEs) demonstrated strong agreement with the observed salbutamol plasma concentrations. The proportion of outliers, determined by assessing the balance of points on each side of the y=x line and the points lying outside the 90% prediction interval, was 4.62%, suggesting an insignificant proportion. Both NPDE and IWRES plots revealed that the residuals are randomly scattered around the horizontal zero-line, indicating that residuals behave as independent standardized normal random variables. Figure 4 illustrates the VPC plot of the final model. It reveals a close alignment between observed (empirical) and predicted (theoretical), within their respective prediction intervals, along with a reduced proportion of outliers. This alignment suggests an adequate fit of the model to the observed data. These diagnostic plots allow for a clearer detection of potential misspecifications in the model. Overall, the developed model is well suited to the salbutamol PK data generated using virtual patients.

### 3.3. External Validation of the Final Structural popPK Model

External validation was conducted by applying the structural model obtained previously to the GSK-derived dataset. The variation of plasma concentration profile over time in the patients enrolled in the clinical study can be visualized in Figure 4, according to stratifications by age, weight, race, and gender. No significant differences are observed, as was noted in the virtual dataset, likely due to the homogeneity of the population. Indeed, this remains a challenge in clinical trials, which has been discussed over the years. Clinical trials are the cornerstone of evidence-based medicine; however, the quality of evidence provided is often poor, particularly regarding participant recruitment. Many clinical researchers tend to perceive heterogeneity as a burden that must be eliminated or controlled through refined statistical approaches to reduce the effects of interindividual variability and achieve consistent results [41,42,43,44]. Nevertheless, the population does not accurately represent the diversity of the overall population. In a 2020 analysis of global participation in clinical trials, the FDA revealed significant disparities among various racial groups: 76% of participants were white, 11% were Asian, and only 7% were black [49]. With the shift in healthcare towards precision medicine, variability in responses across different subpopulations has been recognized. For instance, data from the clinical trial sponsored by GSK predominantly include Caucasian patients, as previously discussed. Several reasons may explain this occurrence. Inclusion and exclusion criteria are usually limited due to ethical and scientific considerations. Including children, for example, in general clinical trials is not very common unless they are pediatric studies designed for that purpose. Participant recruitment alone significantly limits the study, making recruitment of a diverse population in terms of characteristics even more challenging. Attempts to minimize potential confounding variables are also one of the reasons behind the homogeneity of these studies. Ethical and policy issues involving stakeholders may also explain this. Thus, enrolling a homogenous population is not beneficial in any instance for studies seeking to understand the impact of interindividual variability. 

**Figure 5:**
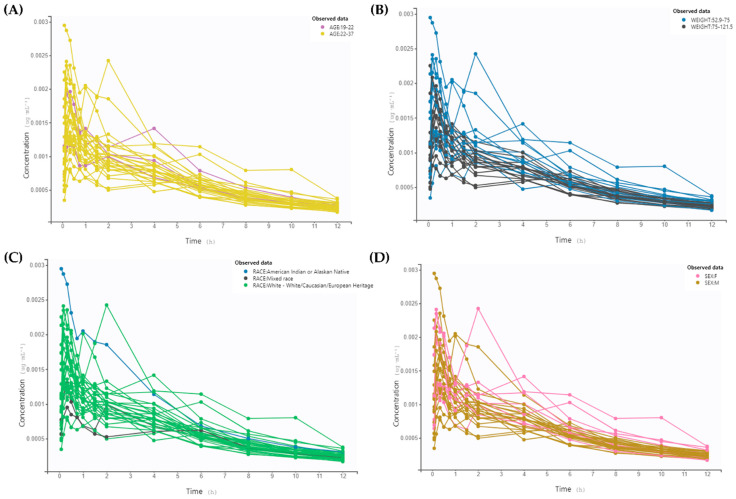
Observed data from the GSK clinical study of plasma salbutamol concentration (μg/mL) through time (h), stratified by (**A**) age, (**B**) weight, (**C**) race, and (**D**) gender.

**Figure 6:**
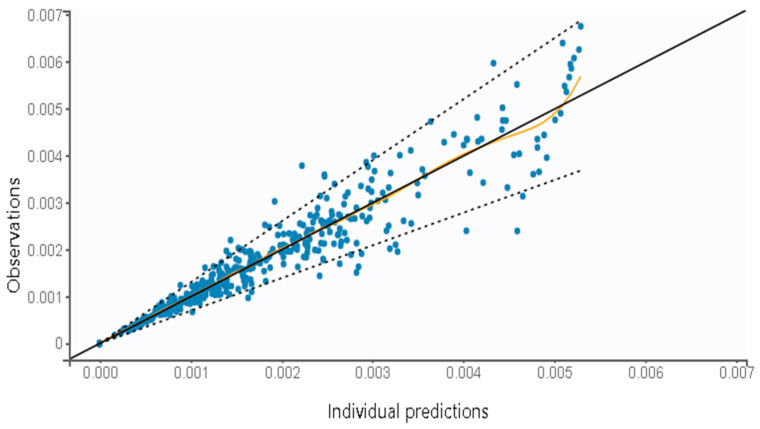
Observed salbutamol concentration vs. individual predictions using conditional mode (EBEs), for the two-compartment model with first-order absorption, no delay, and linear elimination applied to the clinical study dataset for external validation. The dotted line represents 90% prediction interval, the black line refers to y = x line, and the yellow line represents the spline.

**Figure 7:**
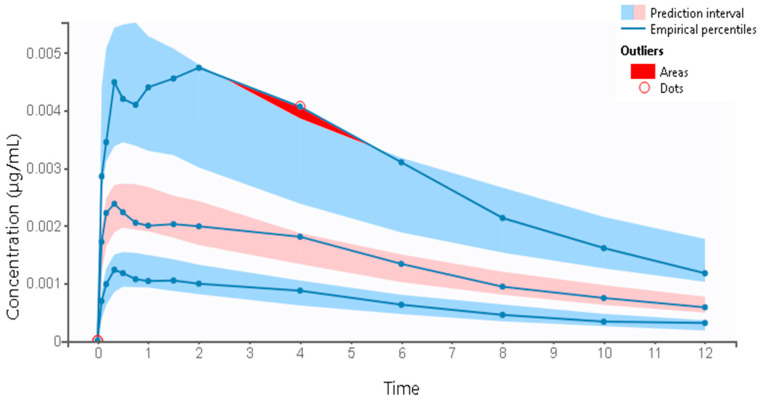
VPC for the two-compartment model with first-order absorption, no delay, and linear elimination applied to the clinical study dataset for external validation.

The two-compartment model with first-order absorption, no delay, and linear elimination was found to fit the data when analyzing diagnostic plots (Figure 5, Figure 6 and Figure 7). Despite an outlier proportion of 7.62% (also visually confirmed in the VPC plot), the model is considered suitable for the data. A −2LL of −7138.63 and a BICc of −7058.17 were obtained. The estimates of the popPK parameters are outlined in Table 4. 

Despite the identified demographic differences between the virtual patients and the clinical trial participants, the range of values in the two datasets is comparable, allowing the validation of the popPK model developed using virtual patients with real-world data. In addition, the need for more heterogeneous clinical trials implies more extensive and costly studies [41]. Therefore, by validating the model developed from synthetic data with real-world data, we can employ this methodology in therapeutic regimen optimization studies. Its advantages are evident, including flexibility, cost-effectiveness, and the ability to simulate diverse patient populations. 

### 3.4. Impact of Covariates on Salbutamol PK Parameters

The developed model revealed the influence of covariates on the PK parameters of salbutamol. Thus, we proceeded to analyze these parameters considering various stratifications for age, weight, gender, and race (Table 5). 

Firstly, the model identified that age influenced C*l* and Q, which, as observed, presented significantly different values when comparing the two subgroups of asthmatic patients (5–22 years and 23–65 years). Q is a parameter that specifies the drug transfer rate between compartments and mainly impacts the drug volume in compartments, which consequently differs significantly between the two subgroups. In general, the V2 is much higher than the volume in V1, suggesting a lower drug availability to target its site of action, increased risk of ADRs due to accumulation in peripheral tissues, or prolonged drug action. At this point, it is not possible to distinguish whether it is a positive or negative effect. In contrast, C*l* is higher in the younger population (5–22 years), potentially indicating a need for closer observation in younger individuals. Maximum efficacy may not be reached, and a dose adjustment may be required. Regarding the absorption of this β_2_-agonist, younger people display increased k_a_, following the PK profile observed in Figure 2A. Indeed, several underlying processes related to aging influence drug disposition, such as reduced first-pass metabolism, decreased organ/tissue mass, increased body fat, and decreased body water content [50]. Concerning the latter one, salbutamol being a hydrophilic drug, with a greater affinity for water, tends to have a lower distribution volume, as confirmed by the presented results.

Weight, in turn, proved to be an important characteristic in the disposition of salbutamol. For individuals weighing over 75 kg (overweight and obese), markedly reduced values were observed for all PK parameters. Regarding the popPK model, relationships between this covariate and C*l*, Q, and V2 were identified. Variations in C*l* may be explained by several factors. For instance, fat accumulation in the liver (the main organ responsible for elimination) may alter hepatic blood flow [51,52]. Changes in enzyme expression of cytochrome P450 (CYP) enzymes, especially CYP2D6 and CYP2C19, may also underlie these decreased values [53]. Further, the discrepancy in drug distribution values is reasonable, considering the hydrophilicity of salbutamol—individuals with higher weight have more adipose tissue. Tissue perfusion may also be reduced in obese patients, impacting Vd (volume of distribution) [52]. The drug absorption rate was also much lower in individuals > 75 kg compared to individuals with normal weight (<75 kg, according to software guidelines). This difference can be attributed once more to changes in blood flow and body composition. 

According to the initial population analysis, gender influences k_a_ and C*l*, which is consistent with the results presented in Table 5. K_a_ and C*l* are both reduced in females. However, C*l* is, indeed, decreased in women (approximately half of the C*l* value displayed in men). These variations may be explained by anatomical differences, namely, weight and total body water volume [4,54]. Consequently, alterations in drug distribution are also expected.

Finally, significant differences were found in Vd and k_a_ between the two racial groups, in contrast to the popPK model results. In particular, American people exhibit higher values for k_a_ and Vd. Among many other factors, well-recognized polymorphisms between racial groups may explain the variation in these PK parameters [55]. Several single-nucleotide polymorphisms (SNPs) have been identified in the coding region of the ABRB2 gene, the gene encoding for the β_2_-adrenoceptor. The most common SNPs result from three missense mutations, one of which involves the substitution of isoleucine (Ile) for threonine (Thr) at codon 164 [56]. According to in vitro studies conducted by Chung et al. [57], this alteration has been associated with decreased receptor binding to the ligand and coupling to Gs proteins in response to different SABAs, namely, salbutamol. This results in a reduction in receptor activity and agonist-stimulated activation. In this regard, the ethnic disparities reflected by the polymorphisms significantly influence the PK parameters of salbutamol, and it is crucial to take them into account when prescribing the drug. 

## 4. Conclusions

This study addresses critical challenges in popPK modeling, particularly regarding data scarcity, incompleteness, and homogeneity in traditional clinical trials. Although the landscape of clinical trials has been changing in recent years, pharmacometricians still face a burden when it comes to PK data. Therefore, by leveraging synthetic data generated through PBPK models, we have overcome these limitations and provided insights into salbutamol’s PK profile across diverse patient populations. External validation using real data from clinical trials is crucial for ensuring the accuracy and reliability of the final models. 

Through this comprehensive analysis, significant associations between individual characteristics and salbutamol’s PK parameters have been identified. Age, weight, gender, and race have indeed a great impact on salbutamol’s ADME processes. This underscores the relevance of personalized dosing strategies, minimizing adverse effects and maximizing therapeutic efficacy. Although the popPK model fails to identify some relationships, these findings revealed great consistency. Further studies using this methodological approach should be conducted, to prove its reliability and capability of predicting interindividual variability in drug responses. 

## Figures and Tables

**Table 1:** Subject characteristics (mean or median ± standard deviation, SD or interquartile range, IR) from virtual dataset and clinical trial.

Characteristics	Virtual Patients (*n* = 32)	Clinical Study Patients (*n* = 30)
Age (years)	20.0 ± 20.0	26.8 ± 4.8
Gender (n, %)		
Female	14, 44%	8, 27%
Male	18, 56%	22, 73%
BMI (kg/m^2^)	21.3 ± 6.6	24.7 ± 3.8
Height (cm)	157.0 ± 30.0	177.8 ± 9.5
Weight (kg)	50.4 ± 21.9	78.9 ± 17.6
Race (n, %)		
American Indian or Alaskan Native	12, 38%	1, 3%
East Asian	20, 62%	ND
Mixed Race	ND	2, 7%
White—White/Caucasian/European Heritage	ND	27, 90%

ND—not determined.

**Table 2:** PK parameters of the virtual and clinical datasets using NCA.

	Virtual Dataset		Clinical Dataset	
Parameter	Geometric Mean	Geometric SD	Geometric Mean	Geometric SD
C*_max_* (μg·mL^−1^)	0.00650	1.61	0.000160	1.35
AUC (μg·h·mL^−1^)	0.00490	1.50	0.00780	1.24
*T_max_* (h)	0.0800	NA	0.310	2.25
C*l* (mL/h)	118	1.50	20689.13	1.21

C*_max_*—maximum observed concentration; AUC—area under the curve from the time of dosing to the last measurable positive concentration; T*_max_*—time of maximum observed concentration; C*l*—clearance; NA—not applicable.

**Table 3:** Estimates of the population pharmacokinetic parameters of the final model for salbutamol DPI formulation.

Parameters	Estimate	RSE (%)
Fixed Effects		
k_a_ (h^−1^)	3.71	2.42
C*l* (L/h)	24.33	20.4
V1 (L)	$0.02\times 10^{-9}$	NaN
Q (L/h)	10.59	1.96
V2 (L)	$0.66\times 10^{-2}$	2.40
Random Effects		
IIV(k_a_)	0.062	36.6
IIV(C*l*)	0.082	13.3
IIV(V1)	10.09	NaN
IIV(Q)	0.045	52.6
IIV(V2)	0.032	30.4
Correlation		
k_a_ and Q	0.89	36.4

RSE: relative standard error; k_a_: absorption constant rate; C*l*: clearance; V1 and V2: the volume of distribution of the compartments one (central), and two (peripheral); Q: intercompartmental clearance; NaN: infinitely large standard error.

**Table 4:** Estimates of the popPK parameters of the final model for salbutamol DPI formulation (using GSK-derived data).

Parameters	Estimate	RSE (%)
Fixed Effects		
k_a_ (h^−1^)	13.55	18.0
C*l* (L/h)	34.93	16.6
V1 (L)	162.93	22.6
Q (L/h)	$1.30\;\times \;10^{-7}$	$2.83\;\times \;10^{6}$ *
V2 (L)	0	$1.35\;\times \;10^{7}$ *
Random Effects		
IIV(k_a_)	0.89	15.4
IIV(C*l*)	0.51	13.3
IIV(V1)	0.41	13.4
IIV(Q)	1.17	$3.88\;\times \;10^{6}$ *
IIV(V2)	0.30	$6.09\;\times \;10^{9}$ *
Correlation		
k_a_ and Q	–0.83	$3.88\;\times \;10^{6}$ *
V1 and C*l*	0.92	3.98

RSE: relative standard error; k_a_: absorption constant rate; C*l*: clearance; V1 and V2: the volume of distribution of the compartments one (central), and two (peripheral); Q: intercompartmental clearance; * values are very large standard errors, potentially suggesting an overparametrization of the model.

**Table 5:** Differences in PK parameters according to the stratification of the virtual population in terms of age, weight, gender, and race. Values presented in geometric mean ± geometric SD.

**Covariates**	**Parameters**				
	**k_a_ (h^−1^)**	**C*l* (mL/h)**	**V1 (mL)**	**Q (mL/h)**	**V2 (mL)**
Age					
5–22	0.0130 ± 13.1	8.77 ± 6.08	$0.540\times 10^{-4}$ ± 508	$0.920\times 10^{-7}$ ± 11.5	0.0460 ± 563
23–65	0.00380 ± 4.62	3.95 ± 3.12	$0.390\times 10^{-4}$ ± 330	$0.140\times 10^{-6}$ ± 124	0.0430 ± 6997
Weight					
17.77–75.00	0.00930 ± 10.7	7.00 ± 5.42	$0.450\times 10^{-4}$ ± 447	$0.140\times 10^{-6}$ ± 59.3	0.067 ± 3320
75.01–105.00	0.00230 ± 1.28	3.17 ± 1.30	$0.540\times 10^{-4}$ ± 297	$0.47\times 10^{-7}$ ± 19.6	0.00440 ± 204
Gender					
Female	0.00610 ± 7.92	5.07 ± 4.01	$0.310\times 10^{-4}$ ± 786	$0.590\times 10^{-7}$ ± 65.0	0.00500 ± 5056
Male	0.00890 ± 11.19	7.37 ± 5.77	$0.660\times 10^{-4}$ ± 228	$0.210\times 10^{-6}$ ± 39.8	0.300 ± 753
Race					
American Indian or Alaskan Native	0.00820 ± 13.8	6.46 ± 4.49	$0.860\times 10^{-4}$ ± 357	$0.270\times 10^{-6}$ ± 65.7	0.0310 ± 1804
East Asian	0.00710 ± 7.62	6.03 ± 5.26	$0.320\times 10^{-4}$ ± 454	$0.690\times 10^{-7}$ ± 42.5	0.0540 ± 3154

k_a_: absorption constant rate; C*l*: clearance; V1 and V2: the volume of distribution of the compartments one (central), and two (peripheral); Q: intercompartmental clearance.

## Data Availability

Data is contained within the article and Supplementary Materials.

## Supplementary Materials

The following supporting information can be downloaded at: https://www.mdpi.com/article/10.3390/pharmaceutics16070881/s1, Table S1: PBPK models and respective individuals’ characteristics; Table S2. Basic popPK models of salbutamol DPI.
