# Supplementary material for: Toward Personalized Salbutamol Therapy: Validating Virtual Patient-Derived Population Pharmacokinetic Model with Real-World Data
Source: Pharmaceutics. 2024 Jun 30;16(7):881. doi: 10.3390/pharmaceutics16070881 (PMC11279662; doi:10.3390/pharmaceutics16070881)
Supplement: Supplementary file 1 [file pharmaceutics-16-00881-s001.zip › pharmaceutics-3007042-supplementary.pdf]

Supplementary Materials: Toward Personalized Salbutamol Therapy: Validating Virtual Patient-Derived Population Pharmacokinetic Model with Re-al-World Data

Lara Marques [1,2,3] and Nuno Vale [1,2,3]

1 PerMed Research Group, Center for Health Technology and Services Research (CINTESIS), Rua Doutor Plácido da Costa, 4200-450 Porto, Portugal; lara.marques2010@hotmail.com
2 CINTESIS@RISE, Faculty of Medicine, University of Porto, Al. Prof. Hernâni Monteiro, 4200-319 Porto, Portugal
3 Department of Community Medicine, Health Information and Decision (MEDCIDS), Faculty of Medicine, University of Porto, Rua Dr. Plácido da Costa, 4200-450 Porto, Portugal
* Correspondence: nunovale@med.up.pt; Tel.: +351-220426537

Table S1. PBPK models and respective individuals' characteristics.

PBPK Model	Population	Gender	Age (years)	Health Status	Height (cm)	Weight (kg)	BMI (kg/m²)	Body Fat (%)
1	American	Male	30	Healthy	176.43	75.0	24.0944	23.41
2	American	Female	30	Healthy	162.20	65.00	24.7065	33.18
3	American	Female	5	Healthy	111.18	20.42	16.5197	21.17
4	American	Female	10	Healthy	142.08	39.43	19.5326	26.17
5	American	Female	20	Healthy	162.71	60.00	22.6630	31.74
6	American	Female	65	Healthy	156.24	60.00	24.5791	33.20
7	American	Male	5	Healthy	111.65	20.06	16.0921	16.17
8	American	Male	10	Healthy	145.23	38.90	18.4432	17.18
9	American	Male	20	Healthy	176.10	75.00	24.1848	23.58
10	American	Male	65	Healthy	173.76	75.00	24.8406	24.08
11	Japanese	Male	30	Healthy	167.16	62.57	22.3924	22.03
12	Japanese	Female	30	Healthy	155.78	50.59	20.8469	26.01
13	Chinese	Female	30	Healthy	159.26	56.03	22.0906	28.07
14	Chinese	Male	30	Healthy	171.00	68.37	23.3816	23.52
15	American	Male	30	Healthy	176.43	85.00	27.3070	26.21
16	American	Male	30	Healthy	176.43	105.00	33.7322	31.80
17	Japanese	Male	5	Healthy	107.80	17.77	15.2915	9.013
18	Japanese	Male	10	Healthy	139.60	32.53	16.6922	12.97

19	Japanese	Male	20	Healthy	169.10	61.89	21.6438	82.33
20	Japanese	Male	65	Healthy	160.34	54.32	31.1289	67.76
21	Japanese	Female	5	Healthy	109.57	18.25	15.2013	36.46
22	Japanese	Female	10	Healthy	138.76	33.26	17.2740	54.06
23	Japanese	Female	20	Healthy	157.79	48.98	19.6725	21.73
24	Japanese	Female	65	Healthy	148.75	47.48	21.5036	21.73
25	Chinese	Female	5	Healthy	106.73	18.13	15.9157	15.26
26	Chinese	Female	10	Healthy	137.39	31.14	16.4971	19.61
27	Chinese	Female	20	Healthy	160.08	52.25	20.3898	26.16
28	Chinese	Female	65	Healthy	153.64	55.42	23.4778	35.45
29	Chinese	Male	5	Healthy	110.49	19.63	16.0796	12.31
30	Chinese	Male	10	Healthy	142.18	35.88	17.7491	16.15
31	Chinese	Male	20	Healthy	172.74	67.14	22.5007	22.46
32	Chinese	Male	65	Healthy	164.88	62.83	23.1117	22.91

BMI, Body Mass Index

Table S2. Basic popPK models of salbutamol DPI.

Project Name	Model Description	Error Model	– 2LL	BICc
PK_S_01	One-compartment model, with no delay, first-order absorption, and linear elimination	Combined 1	–5193.89	–5153.35
PK_S_02	Two-compartment model, with no delay, first-order absorption, and linear elimination	Combined 1	–6480.18	–6420.66
PK_S_03	Two-compartment model, with no delay, first-order absorption, and Michaelis-Menten elimination	Combined 1	–6419.09	–6350.07
PK_S_04	Two-compartment model, with lag time, first-order absorption, and Michaelis-Menten elimination	Combined 1	–6377.79	–6299.28
PK_S_05	One-compartment model, with lag time, first-order absorption, and linear elimination	Combined 1	–5193.78	–5143.74

–2LL, –2 × log likelihood, BICc, Corrected Bayesian Information Criterion
